# Erratum

**DOI:** 10.1080/17482631.2017.1411003

**Published:** 2017-12-20

A number of articles were erroneously published in the issue 12(S2) of *International Journal of Qualitative Studies on Health and Well-being*. Now, these articles are being republished in issue 12(1). The articles that were republished in the issue 12(1) are listed below:

**Empirical studies**

Sandén, U., Harrysson, L., Thulesius, H., & Nilsson, F. (2017). Exploring health navigating design: momentary contentment in a cancer context. *Journal of Qualitative Studies on Health and Well-Being, 12(1)*, doi: 10.1080/17482631.2017.1374809

Holst, H., Ozolins, L-L. Brunt, D., and Hörberg, U. (2017). The learning space—interpersonal interactions between nursing students, patients, and supervisors at developing and learning care units. *Journal of Qualitative Studies on Health and Well-Being, 12(1)*, doi: 10.1080/17482631.2017.1368337

Ørjasæter, K.B., Stickley, T., Hedlund, M., & Ness, O. (2017). Transforming identity through participation in music and theatre: exploring narratives of people with mental health problems. *Journal of Qualitative Studies on Health and Well-Being, 12(1)*, doi: 10.1080/17482631.2017.1379339

**Review article**

Ann-Britt Zakrisson, A.-B. (2017). Symptom-reducing actions: a concept analysis in the context of chronic obstructive pulmonary disease. *Journal of Qualitative Studies on Health and Well-Being, 12(1)*, doi: 10.1080/17482631.2017.1387452

**Empirical studies**

Martínez-Andrés, M., Bartolomé-Gutiérrez, R., Rodríguez-Martín, B., Pardo-Guijarro, M.J. & Martínez-Vizcaíno, V. “Football is a boys’ game”: children’s perceptions about barriers for physical activity during recess time. *Journal of Qualitative Studies on Health and Well-Being, 12(1)*, doi: 10.1080/17482631.2017.1379338

Hwang, A.S., Rosenberg, L., Kontos, P., Cameron, J.I., Mihailidis, A., & Louise Nygård, L. Sustaining care for a parent with dementia: an indefinite and intertwined process. *Journal of Qualitative Studies on Health and Well-Being, 12(1)*, doi: 10.1080/17482631.2017.1389578

Lian, O.S., & Robson, C. “It´s incredible how much I´ve had to fight.” Negotiating medical uncertainty in clinical encounters. *Journal of Qualitative Studies on Health and Well-Being, 12(1)*, doi: 10.1080/17482631.2017.1392219

Loft, M.I., Martinsen, B., Esbensen, B.A., Mathiesen, L.L., Helle K. Iversen, H.K. & Poulsen, I. Strengthening the role and functions of nursing staff in inpatient stroke rehabilitation: developing a complex intervention using the Behaviour Change Wheel. *Journal of Qualitative Studies on Health and Well-Being, 12(1)*, doi: 10.1080/17482631.2017.1392218

Carlsson, I-M., Berg, M., Adolfsson, A. & Sparud-Lundin, C. Reprioritizing life: a conceptual model of how women with type 1 diabetes deal with main concerns in early motherhood. *Journal of Qualitative Studies on Health and Well-Being, 12(1)*, doi: 10.1080/17482631.2017.1394147

Taylor & Francis apologizes for these errors.

